# Juvenile Nasopharyngeal Angiofibroma Presenting with Acute Airway Obstruction

**DOI:** 10.1155/2016/1537276

**Published:** 2016-09-26

**Authors:** Chikoti Wheat, Ryan J. Bickley, Erik Cohen, Danya Wenzler, Nancy Hunter, Donna Astiz

**Affiliations:** ^1^Department of Dermatology, Johns Hopkins University, Baltimore, MD, USA; ^2^Department of Internal Medicine, Morristown Medical Center, Morristown, NJ, USA; ^3^Johns Hopkins University School of Medicine, Baltimore, MD, USA; ^4^Department of Otolaryngology, Morristown Medical Center, Morristown, NJ, USA; ^5^Department of Infectious Diseases, Morristown Medical Center, Morristown, NJ, USA; ^6^Department of Pathology, Morristown Medical Center, Morristown, NJ, USA

## Abstract

We describe a case of a 24-year-old male presenting urgently with a juvenile nasopharyngeal angiofibroma (JNA) with difficulty breathing, inability to swallow, and respiratory distress following throat swelling. The swelling was reduced with administration of dexamethasone and the JNA was surgically resected within 48 hours. This presentation was atypical given the acuity of presentation and the patient's older age.

## 1. Introduction

Juvenile nasopharyngeal angiofibromas (JNAs) are benign nasopharyngeal tumors of high vascularity occurring almost always in prepubertal and adolescent males [1]. They typically present as insidious onset nasal obstruction (80–90%), epistaxis (45–60%), headache (25%), and facial swelling (10–18%) and are most often associated with a history of chronic sinusitis [2–4]. There are various genetic etiologies that have been proposed; however, none of these have been directly linked to nasopharyngeal angiofibromas so that a causal link is yet to be established [5]. In this case report, we present an unusual case of a nasopharyngeal angiofibroma causing obstruction acutely in an adult male.

## 2. Case Report

A 24-year-old male from a local state correctional facility presented to the Morristown Medical Center Emergency Department in Morristown, New Jersey, complaining of throat swelling, difficulty breathing, and inability to swallow beginning four hours prior to presentation. He reported a 6-month history of chronic sinusitis with persistent nasal congestion and clear rhinorrhea for which he had been taking over-the-counter decongestants. He denied any prior symptoms of swelling or obstruction and reported no epistaxis. He denied any recent trauma or exposure to allergenic agents. He admitted to the occasional use of inhaled marijuana but denied any other illicit substance abuse. His only relevant past medical history was latent tuberculosis for which he had been taking moxifloxacin for four months due to exposure to a multidrug resistant strain of tuberculosis prevalent amongst the correctional facility inmates.

On exam, he was moderately dyspneic, with drooling and a muffled voice without adenopathy. Within a few minutes of presentation, he developed progressive respiratory distress and was taken emergently to the operating room where he underwent nasal fiber optic intubation. He was started on combination therapy including dexamethasone, diphenhydramine, fluconazole, and vancomycin for coverage of allergic and infectious etiologies.

**Figure 1:**
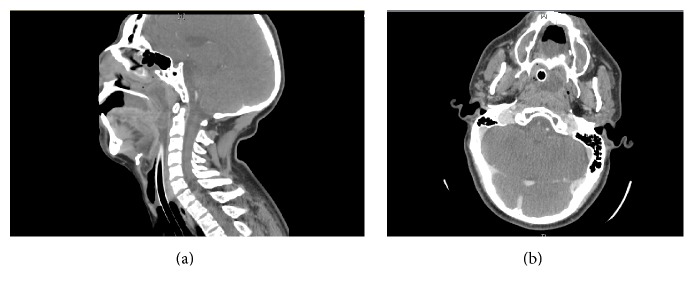
Sagittal (a) and axial (b) contrast enhanced CT images of soft tissue of the neck. Sagittal image shows isodense to hypodense central attenuation with a scattered rim of thin peripheral enhancement (a). The axial image shows the mass located in the right frontal sinus with near complete opacification of the anterior and middle ethmoid air cells and maxillary sinuses with thickening of the pharyngeal mucosa consistent with chronic pharyngitis and sinusitis.

A computed tomography (CT) scan of the neck showed the mass to extend into the oropharynx with surrounding mucosal thickening consistent with chronic sinusitis (Figure 1).

On treatment with dexamethasone, the mass decreased in size, and he underwent transnasal resection 48 hours after presentation. During endoscopic resection, purulent discharge was noted in the middle nasal meatus. Cultures subsequently revealed coagulase negative* Staphylococci* and* Propionibacterium acnes* species.

**Figure 2:**
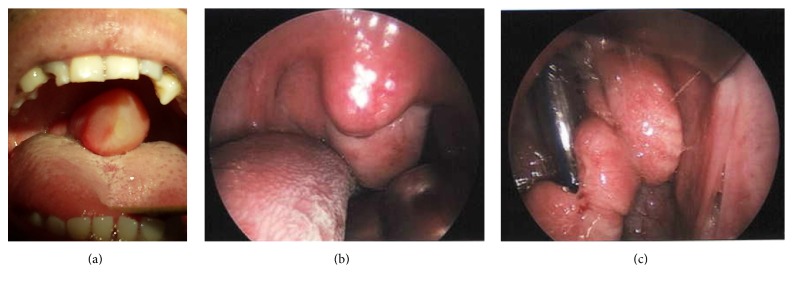
Flesh colored polypoid mass attached to the upper posterior nasopharyngeal wall on initial presentation (a), 36 hours after presentation when patient had been treated with steroids and intravenous antibiotics (b) and following transnasal endoscopic and transoral resection (c).

**Figure 3:**
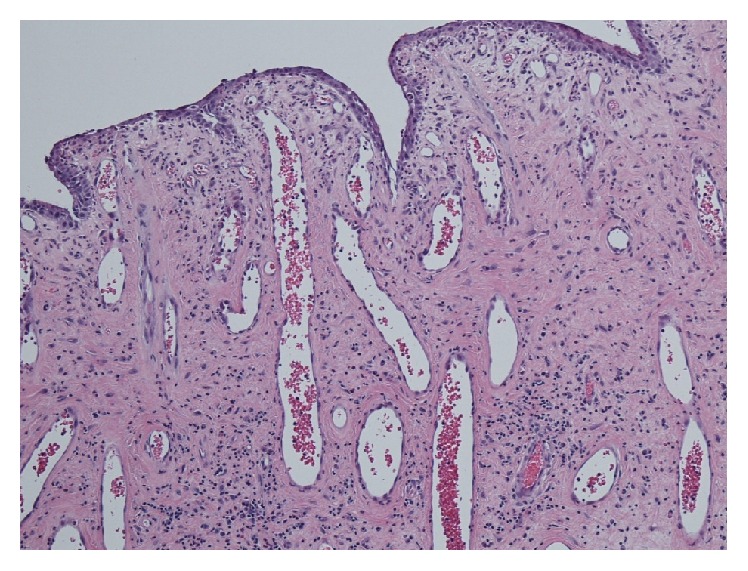
Histologic images highlighting the vascular and stromal components characteristic of a nasopharyngeal angiofibroma. Image showing increased peripheral vascularity.

The resected specimen was a smooth surfaced lobulated mass attached to the upper posterior nasopharyngeal wall (Figure 2). A histologic analysis revealed the mass to be a nasopharyngeal angiofibroma (Figure 3). Microscopic examination of the mass showed high peripheral and reduced central vascularity. The stroma consisted of spindled cells in a sea of randomly arranged collagen with a few scattered vascular channels (Figure 3).

Routine laboratory values appearing on a complete blood count with differential were normal. Additional studies were performed to rule out infectious, allergic, or immunologic etiologies. Both mononucleosis spot test and *β*-hemolytic* Streptococci* blood level test were negative. Lab results were significant for depressed CD4^+^ and CD8^+^ counts (178 and 106, resp.) with a CD4^+^/CD8^+^ ratio of 1.67. An HIV-1/2 antibody test was negative with an HIV-1 RNA load < 75. C1-INH levels were normal. The patient showed normal immunoglobulin levels except for marginally low IgM. He was noted to have EBV test results consistent with past infection (positive EBV capsid IgG, positive EBV nuclear antigen, negative EBV capsid IgM, and negative EBV early antigen). Fungal and acid-fast bacillus cultures were negative.

## 3. Discussion

JNAs commonly occur in patients with histories of chronic sinusitis at least a few months in duration, as is the case with our patient. The acuity in presentation makes this presentation atypical. Though literature reports JNAs to be of insidious onset in adolescent males, there have only been a few reports of these masses occurring in adults [4, 6, 7]. The classic presentation is longstanding unilateral nasal obstruction and recurrent epistaxis neither of which were present in this case. Instead, this patient experienced symptoms that manifested acutely over four hours, although the mass certainly did not present in its entirety over this same timeframe.

Coincidentally, the patient had suppressed CD4^+^ and CD8^+^ T-lymphocyte counts, suggesting a possible causal relationship. Currently, there has been no suggestion of an association between the immune status of the individual and the susceptibility to developing these tumors.

Given that depressed immune function is a risk factor for chronic sinusitis, it is possible that this may also be a risk factor for developing JNA. As of yet, no reports discuss the immune status of patients except for one case report of an HIV positive individual [8]. While our patient was HIV negative with negative viral load, he did have a depressed CD4^+^/CD8^+^ ratio, though not as low a ratio as would be typical for HIV positive patients. Perhaps it is important to consider individual immune status as a risk factor for acute JNAs.

## References

[1] Rubin R., Strayer D. S., Rubin E. (2008). *Rubin's Pathology: Clinicopathologic Foundations of Medicine*.

[2] Tang I. P., Shashinder S., Krishnan G. G., Narayanan P. (2009). Juvenile nasopharyngeal angiofibroma in a tertiary centre: ten-year experience. *Singapore Medical Journal*.

[3] Windfuhr J. P., Remmert S. (2004). Extranasopharyngeal angiofibroma: etiology, incidence and management. *Acta Oto-Laryngologica*.

[4] Mills S. L., Stelow E. B., Hunt J. L. (2012). Tumors of the upper aerodigestive tract and ear. *AFIP Atlas of Tumor Pathology*.

[5] Maniglia M. P., Ribeiro M. E. B., Costa N. M. D. (2013). Molecular pathogenesis of juvenile nasopharyngeal angiofibroma in Brazilian patients. *Pediatric Hematology and Oncology*.

[6] Madhavan Nirmal R., Veeravarmal V., Santha Devy A., Ramachandran C. R. (2004). Unusual presentation of nasopharyngeal (juvenile) angiofibroma in a 45 year old female. *Indian Journal of Dental Research*.

[7] Patrocínio J. A., Patrocínio L. G., Borba B. H. C., De Santi Bonatti B., Guimarães A. H. B. (2005). Nasopharyngeal angiofibroma in an elderly woman. *American Journal of Otolaryngology—Head and Neck Medicine and Surgery*.

[8] Landonio G., Nosari A., Oreste P., Cantoni S., Cattaneo D., Ghislandi E. (1993). Aggressive course of angiofibroma in an HIV-positive patient. *Tumori*.

